# An Evaluation of an eHealth Tool Designed to Improve College Students’ Label-Reading Skills and Feelings of Empowerment to Choose Healthful Foods

**DOI:** 10.3389/fpubh.2017.00359

**Published:** 2018-01-11

**Authors:** Lisa M. Soederberg Miller, Carolyn A. Sutter, Machelle D. Wilson, Jacqueline J. Bergman, Laurel A. Beckett, Tanja N. Gibson

**Affiliations:** ^1^Department of Human Ecology, University of California Davis, Davis, CA, United States; ^2^Family Resiliency Center, University of Illinois, Urbana-Champaign, Urbana, IL, United States; ^3^Department of Public Health Sciences, University of California Davis, Davis, CA, United States; ^4^Nutrition Department, University of California Davis, Davis, CA, United States

**Keywords:** eHealth tools, nutrition label reading, empowerment, skill development, college students

## Abstract

**Objective:**

College students are at risk for poor dietary choices. New skills can empower individuals to adopt healthful behaviors, yet eHealth tools designed to develop food-choice skills, such as label-reading skills, are uncommon. We investigated the effects of web-based label-reading training on college students’ perceptions of healthful food-choice empowerment.

**Methods:**

Students completed label-reading training in which they practiced selecting the more healthful food using nutrition labels. We examined improvements in label-reading accuracy (correct healthfulness decisions) and perceptions of empowerment, using a 6-item scale. Repeated measures ANOVAs and paired-samples *t*-tests were used to examine changes in accuracy and empowerment across the training session.

**Results:**

In addition to increases in label-reading accuracy with training, we found increases in healthful food-choice empowerment scores. Specifically, the proportion of correct (i.e., more healthful) food choices increased across the three blocks of practice (*p* = 0.04) and food-choice empowerment scores were about 7.5% higher on average after training (*p* < 0.001).

**Conclusion and implications:**

Label-reading training was associated with increased feelings of empowerment associated with making healthful food choices. Skill focused eHealth tools may offer an important avenue for motivating behavior change through skill development.

## Introduction

College years are a time of risk for weight gain and for establishing food-choice patterns (1, 2). Nutrition labels, which contain information needed to follow Dietary Guidelines for Americans, could be important information sources for college students as they begin to choose foods independently. Although some students report using nutrition labels for health reasons, weight control, or to find specific nutrition information (3, 4), many report they do not use labels. A large-scale study showed that 55% of students reported that they had never read, heard of, or been taught information about how to use food labels (5). Underutilization of labels is also due to lack of exposure and understanding (5–7).

A range of approaches has been used to encourage college students to use nutrition labels. One approach involves providing point-of-purchase labels in places, such as dining halls, convenience stores, and campus vending machines, to help students (8–11). Although labeling draws attention to the nutrition information, some students may not understand how to use the information to choose foods. Another approach involves mobile applications (apps) that interpret nutrition information. For example, traffic-light labeling systems may use green for to-be-encouraged foods, such as those low in sugars, and red for to-be-discouraged foods, such as those high in saturated fat (12–14) when the individual scans the label (e.g., at a grocery store). However, relatively less attention has been paid to teaching students to read nutrition labels, an approach that attempts to place the “know-how” in the student, rather than rely on an app. Some work in this area has shown that label-reading training can be effective using a brief video and pocket card (15) as well as a web-based tool that develops label-reading skills through focused practice (16).

The Information-Processing (IPR) framework argues that ability and motivation interact and evolve with practice and skill development (17). Thus, training that includes practice reading nutrition labels may promote motivation surrounding food-choice skills. Although past research has shown positive associations among food-choice skills and motivational factors, such as attitudes and beliefs (3, 5, 18–22), it is less clear if the acquisition of new label-reading skills increases motivation. Empowerment, a form of motivation, refers to perceptions of enhanced capacity to make informed decisions and actions affecting one’s health (23–25). Empowerment can result from teaching individuals with chronic conditions how to perform self-care tasks (26) and parents of young children how to make healthful choices for their family (27, 28).

In the present study, pre–post changes in food-choice empowerment were examined as a result of label-reading training using a web-based tool that provides intense, focused practice (16). As in past research, the training occurred in a single session (15, 16). Students’ usability perceptions of the web-based training were assessed to gain information about users’ impressions of the new nutrition label training approach. An investigation of empowerment resulting from a web-based training tool is needed to understand better the power of skill acquisition on feelings of empowerment. The work could also inform research on ways to increase participant engagement in eHealth and mHealth interventions (29, 30).

## Materials and Methods

Self-reported food label use was assessed to determine whether students’ prior experience affected training outcomes. The question, “How often do you use food labels to make choices regarding healthful foods to buy, especially when purchasing the food for the first time?” (31) was used and responses were made by positioning a marker on a continuous sliding scale from 0 (never) to 100 (always).

### Training

The training consisted of a 20-min narrated slide tutorial of general information about nutrition (e.g., description of major nutrients and their role, energy, nutrient sources in foods, and diet–health relations) and an overview of food label information (e.g., the different nutrient types and metrics). This was followed by an orienting task in which participants were asked to locate a specific piece of information on the food label (nutrition label, ingredient list, or front of package).

The focused-practice part of the training consisted of comparing two labels to determine which was the healthier choice within the context of one’s daily diet (16). The training, grounded in the IPR framework and research on skill acquisition showing that focused practice can lead to automatic processing that is efficient (fast and accurate) (17, 32), has been shown to improve label-reading speed and accuracy (16). Label pairs were designed to differ a great deal in one or two nutrients but only a little in other potentially distracting nutrients so that, across pairs, the more healthful choice is determined by a variety of different nutrients. With practice, the task teaches individuals to identify meaningful nutrient differences between two foods while ignoring insignificant differences. Comparisons were organized into three practice blocks of 24 comparisons, with immediate feedback (i.e., correct or incorrect) provided after each comparison and total scores provided at the end of each block.

### Measures

#### Label-Reading Accuracy

Label-reading accuracy was assessed for each of the three practice blocks as the percentage of correctly identified healthful choices out of 24 label comparisons.

#### Healthful Food-Choice Empowerment

The healthful food-choice empowerment measure, designed specifically for this study, consisted of six items (see Table 1) assessing perceptions of ability and willingness to use nutrition information and choose healthful foods, as well as current and desired understanding level of nutrition information. Participants were instructed to indicate their perceptions by positioning a marker on a continuous sliding scale from 0 to 100 with anchors labeled *low* and *high*, respectively. The items, used at pre- and post-test, showed good internal consistency with standardized Cronbach’s alpha (33) (a measure of reliability that evaluates each item in relation to the total scale) of 0.87 and 0.88, respectively, and were averaged to form an overall pre- and post-test score.

**Table 1:** Means and changes (unadjusted) in healthful food-choice empowerment items and average score from pre- to post-training in 44 university students.

Empowerment items	Pre-mean (SD)	Post-mean (SD)	P*
My ability to select healthful foods is	68 (21)	74 (19)	0.063
My willingness to select healthful foods is	67 (23)	75 (21)	0.009
My ability to use nutrition labels to select healthful foods is	66 (23)	82 (13)	<0.001
My willingness to use nutrition labels to select healthful foods is	67 (26)	79 (19)	<0.001
My current level of understanding nutrition principles and healthful dietary practices is	64 (19)	77 (13)	<0.001
My desired level of understanding nutrition principles and healthful dietary practices is	88 (13)	88 (18)	0.733
Empowerment, overall score	73 (15)	80 (13)	**<0.001**

*Paired t-test.

*Scale = 1–100 with anchors labeled low to high, respectively*.

*Significance values in bold*.

#### Usability

We assessed user experience with the e-training tool in four areas. Usability of the tool was assessed in terms of (1) ease of use, (2) usefulness, and (3) enjoyableness (34). Instructions were, “Your feedback is important to us! Please select the responses that best fit your opinion.” Participants were prompted with “The tasks were____” and asked to position a marker on a 0–100 scale with anchors: *difficult/easy to complete; boring/enjoyable to complete; not at all useful/very useful*, respectively. In addition, participants were asked to indicate whether they felt their label-reading skills would improve with more practice (16) with anchors *not at all likely to improve/very likely to improve*.

### Procedure

Ethical approval was obtained from the university’s Institutional Review Board (697420) and all participants provided informed written consent. Participants completed a brief survey of demographic information, including food label use and healthful food-choice empowerment, which was followed by the nutrition tutorial, orienting task, and label-reading comparison task. The sample comparison that students received immediately prior to beginning the comparison task was manipulated such that half the participants received only correct/incorrect feedback on the sample and half received correct/incorrect feedback plus a detailed illustration (using highlighting) indicating the large nutrient differences between the foods (explicit feedback). Participants were assigned alternately to treatment and control conditions as they enrolled. At the end of the training, participants completed the healthful food-choice empowerment post-test and the usability survey. All tasks were completed *via* an online portal in under 2 h.

### Statistical Methods

In addition to calculating demographic variable means, repeated measures ANOVA was conducted to test for practice block effects on accuracy with explicit feedback, sex, and self-reported food label use as covariates. Similarly, a repeated measures ANOVA was used to test for pre/post effects on overall empowerment with the same set of covariates. Paired *t*-tests were used to test for overall differences in pre/post empowerment and item level differences in empowerment. Usability perceptions were examined with descriptive statistics. A *p*-value of 0.05 was used to assess significance. Analyses were performed using SAS^®^ software version 9.4.

## Results

### Sample

Forty-four college students (18 males and 26 females) were recruited through an online subject recruitment system and received college credit for participation as part of an introductory psychology course. Participation eligibility included being a fluent speaker of English and between the ages of 18 and 80. There were no other restrictions on participation. Participants were on average 19 years old (ranging from 18 to 26) with 14 years of education. The sample was 59% female, with 23% reporting they were of Hispanic ethnicity. On average, students reported using food labels a little more than half the time, based on a mean of 56.1 (SD = 29.7) on a self-reported frequency (0 = never; 100 = always) of food label use measure. The treatment and control groups did not differ in terms of sex (*p* = 0.12), age (*p* = 0.41), education (*p* = 0.22), Hispanic ethnicity (*p* = 0.99), or food label use (*p* = 0.75).

### Label-Reading Accuracy

The dependent variable was the proportion correct for each of the three label-reading practice blocks. The repeated measures ANOVA revealed significant improvement from Block 1 to Block 2 (87 vs 90%, *p* = 0.021) and from Block 1 to Block 3 (87 vs 90%, *p* = 0.044), but not between Block 2 to Block 3 (90 vs 90%, *p* = 0.98). Males performed on average about 6% better than females in Block 1 (*p* = 0.03), but this effect disappeared in Blocks 2 and 3 (*p* = 0.81 and 0.77, respectively). There was no significant effect from food label use or the explicit feedback (*p* = 0.35 and 0.13, respectively).

### Healthful Food-Choice Empowerment

**Figure 1:**
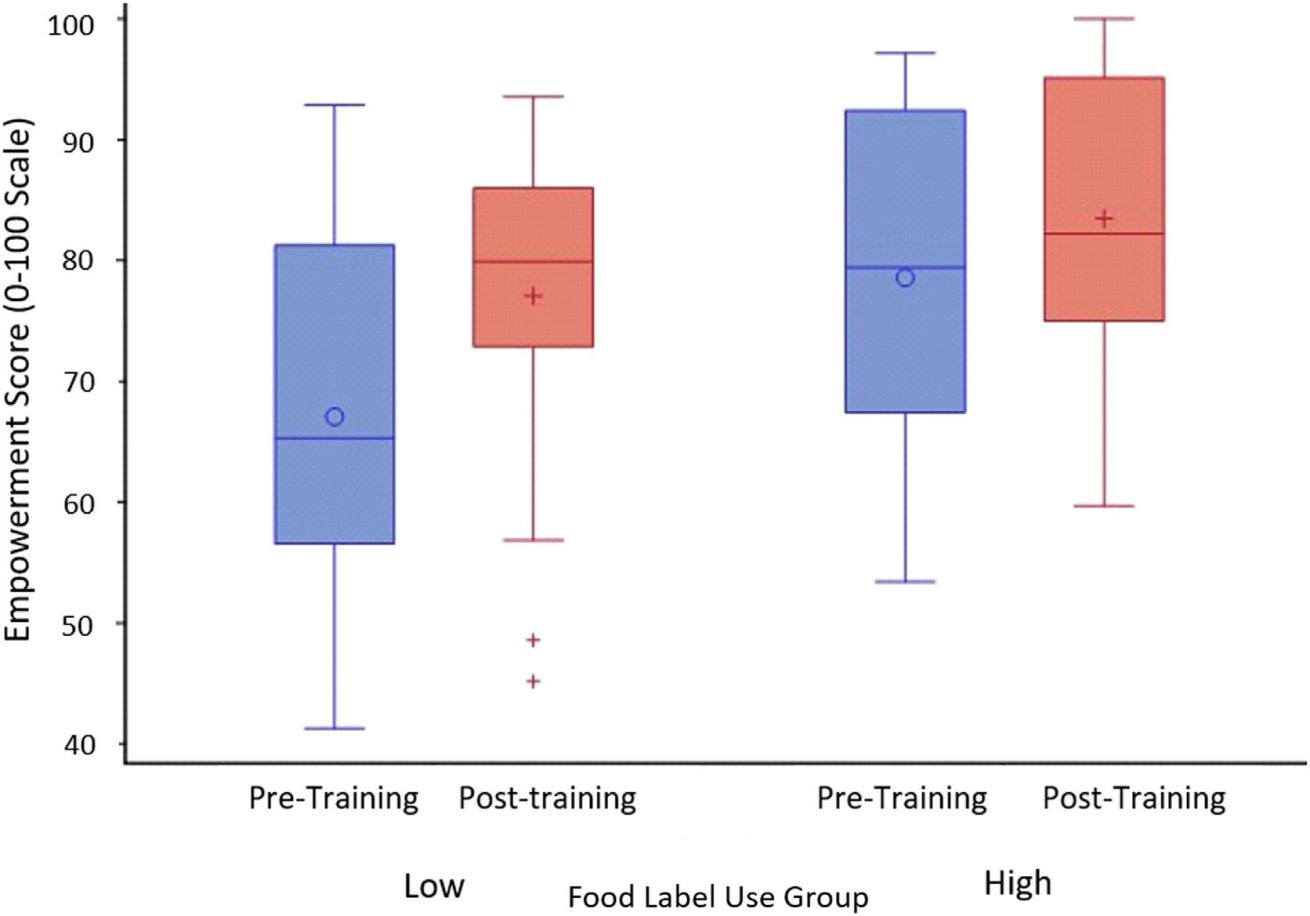
Box and whisker plots for pre- and post-training empowerment scores for low- and high-food label use groups. High vs low food label use was defined by a median split, yielding 22 participants in each group. Boxes represent the 25th and 75th percentiles. Whiskers represent the range from minimum to maximum of observed data. *P*-values from the mixed effects ANOVA model were *p* = 0.07 for time by food label use interaction and *p* < 0.001 for the main effect of training (time).

The paired *t*-test revealed that students showed significant increases in their overall food-choice empowerment scores from pre- to post-training (*p* < 0.001), scoring about 7.5% higher on average after training. Results also showed that self-reported food label use was associated with a significantly higher empowerment at pre- (*p* < 0.001) and post-training (*p* = 0.02). As shown in Figure 1, there was a trend for a greater effect of training on empowerment for those with lower food label use compared to those with higher, but the time by food label use interaction was non-significant (*p* = 0.07). There were also no significant effects due to explicit feedback (*p* = 0.12), sex in either pre- (*p* = 0.35) or post- (*p* = 0.10), or time by sex interactions (*p* = 0.35).

Table 1 contains the means and change coefficients for each empowerment item. One item, “desired level of understanding nutrition principles and healthful dietary practices,” failed to increase significantly with training. The pre-test mean (88) was relatively high for this item, however, which could have limited the possibility of improvements. Another item, “ability to select healthful foods,” showed a trend toward improvement but was non-significant (*p* = 0.06). The remaining four items increased significantly (*p* < 0.001).

### Usability

Generally, participants found the training tool easy to use [average score 87.1 (SD = 16.0)] and felt their label-reading skills would continue to improve with more practice [average score of 80.2 (SD = 24.3)]. Students rated the training tool somewhat lower on usefulness [74.2 (22.1)] and enjoyment [67.5 (22.7)].

## Discussion

Results from the present study show that college students’ feelings of empowerment increased with label-reading skills and were associated with self-reported food label use. Gains in label-reading skills may foster feelings of motivation, competence, and willingness to take control of healthful food-choice behaviors. The notion that nutrition knowledge, motivation, and label use are interrelated is not new (3, 5, 18–20, 22, 35), and both knowledge and motivation have been shown to support nutrition label use (21). Moreover, past research also has shown that empowerment can result from teaching individuals with chronic conditions how to perform self-care behaviors (26) and teaching parents of young children how to make healthful choices for their family (27, 28). Findings from the present study add to these areas of research by indicating that empowerment can result from developing nutrition label-reading skills among college students. The web-based platform has wide-reach potential and provides easy access for college students, so could offer an important leverage point to improve nutrition knowledge, skills, and empowerment at a time when young adults are beginning to make autonomous dietary choices.

### Limitations

Although the sample size was comparable to other studies targeting behavior change (36, 37), students participated for credit, limiting the diversity of the sample. A larger, more diverse (e.g., age, education, region of the country) sample would have allowed examination of within group differences in factors such as prior experience using nutrition labels and socio-economic status. Still, the sample size was sufficient to show that label-reading skills and empowerment increased with training, with suggested greater effects for those less accustomed to using labels. However, it remains possible that a different group of students may be less inclined to engage in the web-based practice task. Another limitation of the present study was generally high performance in label-reading accuracy at pre-test. A sample including a greater proportion of students with little or no prior experience with food labels would be informative. Also, follow-up assessments would be important in future research to determine the extent to which skills are maintained and applied to food choices in the short and long term.

## Implications for Research and Practice

Findings from the present study demonstrate that nutrition label-reading training, over the course of a single session, increases accuracy in food label comparisons and feelings of empowerment surrounding healthful food choice. The implication is that individuals benefit from gaining new skills in ways that extend beyond the skill itself. A diet app that provides answers to healthfulness of various foods can be a simple way to address a specific point-of-purchase decision. However, learning a skill enables the individual to select more nutritious food choices without reliance on an app and increases empowerment, operationalized as perceptions of ability and willingness to use these skills and seek healthier foods. The added benefit of increased empowerment is especially important for training with high-tech approaches, as prior research has noted low engagement as an issue in electronic-based training and interventions (29, 30). With increased empowerment to make healthful choices, individuals may be more likely to engage with training and continue to improve skills. Because of the web-based platform, the tool has potential for wide reach as a stand-alone tool or to supplement personalized nutrition coaching by reinforcing concepts, facilitating discussion, and providing support for grocery shopping and other food-choice skills (e.g., meal planning, budgeting, and cooking).

## Ethics Statement

The study was carried out in accordance with the recommendations of Association for the Accreditation of Human Research Protection Programs, Inc. (AAHRPP) with written informed consent from all subjects. All subjects gave written informed consent in accordance with the Declaration of Helsinki. The protocol was approved by the university’s Institutional Review Board (697420).

## Author Contributions

LM was responsible for conceptualizing the project, study design, data collection, data analysis, interpretation of finding, and drafting and revising the manuscript. CS assisted with interpretation of findings as well as writing and revising the manuscript. LB helped conceptualize the project, interpret finding, and edit the manuscript. MW conducted statistical analyses and wrote and revised sections of the manuscript. JB aided with the production of the tutorial and assisted with interpretation of findings. TG assisted with study design, data collection, and drafting parts of the manuscript.

## Conflict of Interest Statement

The authors declare that the research was conducted in the absence of any commercial or financial relationships that could be construed as a potential conflict of interest.
